# Three-Month Follow-Up of the Post-COVID Syndrome after Admission to a Specialised Post-COVID Centre—A Prospective Study Focusing on Mental Health with Patient Reported Outcome Measures (PROMs) †

**DOI:** 10.3390/ijerph21081076

**Published:** 2024-08-16

**Authors:** Isabel Cecil Schäfer, Johannes Krehbiel, Werner Adler, Andrea Borho, Regina Herold, Brigitte Greiner, Miriam Reuner, Eva Morawa, Yesim Erim

**Affiliations:** 1 Department of Psychosomatic Medicine and Psychotherapy, University Hospital of Erlangen, Friedrich-Alexander University Erlangen-Nürnberg (FAU), 91054 Erlangen, Germany; johannes.krehbiel@uk-erlangen.de (J.K.); werner.adler@fau.de (W.A.); borho.andrea@yahoo.de (A.B.); regina.herold@uk-erlangen.de (R.H.); brigitte.greiner@uk-erlangen.de (B.G.); miriam.reuner@fau.de (M.R.); eva.morawa@uk-erlangen.de (E.M.); yesim.erim@uk-erlangen.de (Y.E.); 2 Post-COVID Centre, University Hospital Erlangen, 91054 Erlangen, Germany; 3 Department of Medical Informatics, Biometry and Epidemiology, Friedrich-Alexander University Erlangen-Nürnberg (FAU), 91054 Erlangen, Germany

**Keywords:** post-COVID syndrome, PASC, mental health, psychological trajectory, long COVID, PROMs

## Abstract

Background and objective: The impairments and duration of PASC (post-acute sequelae of COVID-19) symptoms in mental health have, to date, not been comprehensively examined. Our objective is to provide longitudinal data on the mental health of Post-COVID patients and to identify risk and protective factors associated with a severe or prolonged course. Methods: The mental health of 265 Post-COVID patients of the outpatient Post-COVID centre of the University Hospital Erlangen was assessed 17.1 (T0) and 22.5 months after infection (T1). An online survey with validated questionnaires for Post-COVID symptoms (Post-COVID Syndrome Score), depression (Patient Health Questionnaire-9), somatic symptoms (Patient Health Questionnaire-15), anxiety (Generalized Anxiety Disorder-7), fatigue (Fatigue Severity Scale) and Post-Exertional Malaise (PEM) (DePaul Post-Exertional Malaise Screening) was conducted in the home environment. Results: In total, 80% of patients experienced severe PASC at follow-up. Clinically relevant symptoms of depression, persistent somatic symptoms, anxiety and fatigue were reported by 55.8%, 72.5%, 18.9% and 89.4% of patients, respectively. Depressive, anxiety and somatic symptom severity decreased significantly over time; fatigue and PEM remained at an unchanged high level. The risk factor for higher depression scores was older age; prior psychiatric illness treated with psychotherapy was associated with more severe depressive, somatic, anxiety and PASC symptoms. PEM symptoms were significantly associated with longer duration between acute infection and initial presentation in the Post-COVID centre. Conclusions: Our findings align with previous research, claiming severe mental health symptoms in PASC syndrome, lasting for months after infection. In-depth assessment of risk and protective factors for the mental health implications of PASC is needed for the planning of health services and disease prevention.

## 1. Introduction

### 1.1. Post-COVID Syndrome

According to the World Health Organization, around 10–20% of patients afflicted with the SARS-CoV-2 virus still suffer from symptoms 3 months after infection [1]. As long as there is no other explanation for these prolonged symptoms, this condition is referred to as Post-COVID or PASC (post-acute sequelae of COVID-19) [1,2,3].

While fatigue and dyspnoea are the most common symptoms [4,5], many patients also experience neuropsychiatric or psychological symptoms encompassing cognitive impairment, memory loss, brain fog, anxiety and depression [5,6,7,8].

The precise pathophysiology of PASC remains incompletely understood. Common hypotheses include autoimmunity and inflammation, potential viral tissue reservoirs or alterations in the gut microbiome [4,9,10]. What has become evident is that COVID-19 and PASC affect multiple organs, extending beyond the respiratory tract [11].

Risk factors for PASC include female sex, older age, pre-existing comorbidities, neuropsychiatric diseases, longer hospital stays and high BMI [4,5,12]. Some studies suggest that the severity of acute COVID-19 is not associated to PASC onset [13,14], while others postulate a higher PASC prevalence after severe illness [5,10].

### 1.2. Mental Health Impairments in PASC

PASC not only manifests in physical symptoms but also exerts an impact on the individual’s mental health. Several analyses have observed that 14.5% to one-fifth of individuals recovering from an infection exhibit mental health problems [15,16].

Common psychological symptoms in PASC include depression (12%; 4–31%), anxiety (23%; 6.5–63%), concentration difficulties (22%) and fatigue (37%; 28–87%) [6,17,18]. Other symptoms include Post-Exertional Malaise (PEM) (89%) [19], somatization (37.5%) [20] and Post-Traumatic Stress Disorder (12.1% to 46.9%) [17]. Due to the highly heterogeneous patient collectives, numbers vary significantly.

Several risk factors for the development of psychological symptoms have been identified, a prior mental disorder emerging as the most significant [4,12]. Further, females, patients admitted to ICU during acute infection and younger people exhibited a higher likelihood of experiencing mental health problems [6,8,17]. A potential general protective factor for psychiatric Post-COVID symptoms is vaccination [12].

Despite limited long-term longitudinal research, several studies support the hypothesis of a high symptomatic burden with minimal improvement within 3–12 months [21,22,23,24]. One cross-sectional meta-analysis found that patients still suffer from somatization (37.5%), fatigue (26.2%), anxiety (18%) and depression (12.6%) 12 months after infection [20]. Further, Guillen-Burgos conducted a comparative analysis among 1565 patients, assessing their status at 12 and 24 months, revealing only a slight decrease in depression from 33.6% to 21.79% and anxiety from 27.90% to 16.55% [25]. Studies with longer surveying periods show persisting mental health symptoms up to 24 months after infection [26,27].

This persisting impairment on everyday life results in 58% of PASC patients reporting a poorer quality of life than before the infection [15]. In particular, symptoms like fatigue and PEM were identified as severely impacting mental health [15]. Both PEM and fatigue persisted over a 12-month period without significant improvement, and some studies even showed a worsening over time [20,28,29,30].

The question of PASC duration and whether PASC follows a self-limiting course, like some other post-viral syndromes [31], remains unanswered, especially concerning the duration and alterations in the mental health and psychological trajectory.

### 1.3. Research Objectives

This study endeavours to offer longitudinal data concerning the mental health trajectory of Post-COVID patients, given the current scarcity of research therein. Our objectives were firstly to examine the trajectory of PASC symptoms and mental health outcomes, i.e., whether PASC follows a self-limiting course, and secondly to identify both risk and protective factors associated with a more severe symptomatology in PASC patients.

## 2. Material and Methods

### 2.1. Participants and Data Collection

This research took place in the interdisciplinary outpatient Post-COVID centre of the University Hospital Erlangen. Individuals experiencing ongoing symptoms after confirmed COVID-19 infection were referred by their general practitioners with the suspected diagnosis of PASC. These patients were consecutively recruited for this study between December 2022 and February 2024.

Inclusion criteria were a minimum age of 18 years, confirmed COVID-19 infection, and symptom persistence for 3 months without another explanation as observed by the general practitioners. Exclusion criteria encompassed Post-Vaccination Syndrome and a high symptomatic burden that prevented participation.

During their clinical appointment (T0), patients underwent psychological/psychosomatic evaluation via in-depth interviews with mental health professionals (physicians) and extensive neurocognitive/neuropsychological testing [7]. Additionally, participants completed different validated questionnaires (Patient Reported Outcomes Measures, PROMs) at home, using an online survey.

Using an online survey with PROMs allowed for a comprehensive assessment and monitoring of the diverse symptoms of PASC patients at T0 and T1, especially considering that some patients could not attend two appointments due to their physical impairments. Conducting the survey at home saved both time as well as medical capacity.

Moreover, data concerning sociodemographic, health behaviour, pre-existing (psychiatric/psychosomatic) diseases and pain were collected.

A specialist of internal medicine reviewed important findings, e.g., echocardiograms and current laboratory results, and examined the patients physically. This process helped rule out immunological diseases that could have been the cause of the patients’ complaints.

Based on these interdisciplinary diagnostics, patients received classification regarding PASC and their symptom severity and recommendations regarding possible treatment options.

The follow-up survey (T1) started in October 2023 and lasted until February 2024. It was conducted at least 3 months after first presentation to the centre (T0). Taking into consideration that the follow-up study (T1) started in October 2023, the maximum time span between T0, which started in December 2022, and T1 was 10 months.

The same questionnaires were employed at T0 and T1. The survey took approximately 45 min and was conducted in the home environment without clinical presentation.

Patients were invited to participate via letter and E-Mail. Throughout a four-week period, non-responders received one weekly reminder, via E-Mails (3 times) and one phone call.

This study was approved by the ethics committee of the Friedrich-Alexander-University Erlangen-Nürnberg, (T0: 22-443-B, T1: 22-444_3-B) and all participants gave their written informed consent.

### 2.2. Measures/Variables

#### 2.2.1. Sociodemographic and Health-Related Data

The collected sociodemographic variables comprised sex, age, marital status, number of children, education level and current employment status at baseline and follow-up. Further, information regarding health behaviour, such as smoking habits, BMI and the presence of pre-existing illnesses was gathered.

The following PROMs were used at T0 and T1:

#### 2.2.2. PASC Symptoms

PASC symptoms were assessed using Post-COVID Syndrome Score (PCS-S) [32], which consists of 12 dichotomised questions covering various symptoms: ageusia or anosmia, fatigue, lack of physical resilience, joint or muscle pain, throat/nose/ear discomfort, lung/breathing difficulties, cardiac symptoms, intestinal symptoms, neurological complaints, dermal problems, signs of infection and sleeping disorders. Each answer weighs differently, resulting in a scale from 0 to 59. Cut-off values are as follows: no/mild PCS ≤ 10.75, moderate PCS > 10.75 to ≤26.25 and severe/relevant PCS > 26.25. Cronbach’s alpha was 0.66 at T0 and 0.67 at T1.

Additionally, information regarding the acute COVID-19 infection was collected: the date of the acute infection and the course of infection (asymptomatic infection, at home or outpatient therapy, symptomatic infection requiring clinical therapy, symptomatic infection requiring ICU admission).

Further, information about vaccination and proof of infection by positive PCR was gathered.

#### 2.2.3. Mental Health Symptoms

##### Generalized Anxiety Disorder-7 (GAD-7)

Symptoms of anxiety were assessed using the GAD-7, with total scores from 0 to 28. A score of ≥10 indicates clinically significant symptoms of anxiety [33]. Cronbach’s alpha was at T0 0.87 and at T1 0.89.

##### Patient Health Questionnaire-9 (PHQ-9)

Depressive symptoms were measured with the PHQ-9. Sum scores range from 0 to 27 points, whereas clinically significant depression can be assumed at ≥10 points [34]. Cronbach’s alpha at T0 was 0.78 and at T1 0.78.

##### Patient Health Questionnaire-15 (PHQ-15)

The PHQ-15 was used to assess the severity of somatic symptoms. A cut-off value of ≥10 points hints at relevant somatization [35]. Cronbach’s alpha at T0 was 0.80 and at T1 0.81.

##### The Fatigue Severity Scale (FSS)

This questionnaire consists of nine statements to assess fatigue with a Likert scale, between 1 and 7 (range: 9–63). A mean score of ≥4 indicates clinically relevant fatigue [36]. Cronbach’s alpha was 0.94 at T0 and 0.95 at T1.

##### DePaul Post-Exertional Malaise (PEM) Screening

Post-Exertional Malaise, meaning complete and disproportionate exhaustion induced by physical or mental activities, was assessed with this questionnaire. Patients answered questions regarding the severity of exhaustion after different activities, as well as the frequency of this occurring [37]. Cronbach’s alpha was 0.71 at T0 and 0.62 at T1.

##### Psychotherapeutic Treatment

The follow-up survey queried participants about psychotherapeutic treatments before developing PASC using the question, “Have you ever been in psychotherapy/psychosomatic therapy/psychiatric therapy before your Post-COVID symptoms?”

##### Cardiological Data

Patients presenting at the Post-COVID centre were required to provide echocardiography results carried out after their COVID-19 infection. These echocardiograms were analysed to assess their physical health and detect potential cardiac damage. The analysis was performed with an algorithm displayed in Supplement S1.

##### Other Data

Additional data were collected through validated questionnaires and are reserved for upcoming publications. These include assessments of social support, loneliness, insomnia, stress, pain, quality of life and coping with illness.

Moreover, patients were asked about the various treatments they had utilized since T0 and how these treatments affected their symptoms.

### 2.3. Data Analysis

SPSS V. 28 (IBM Corporation, Armonk, NY, USA) was utilized to analyse the data. Descriptive statistics including means, standard deviations, ranges and frequencies were computed. The prevalence of mental health conditions was determined using established cut-off values. The paired *t*-test was employed to assess differences in examined variables between T0 and T1. The one sample *t*-tests were performed to compare the study cohort to the general population. The McNemar test was used to establish the prevalence of mental health impairments over validated cut-off values. We examined the relationship between independent variables at T0: sex, age, dichotomized BMI, smoking, echocardiography, occupation, psychological condition, relationship, time interval, education, course of disease, number of vaccinations and time point, and the dependent variables PHQ-9, PHQ-15, GAD-7, DSQ-PEM, FSS and PSC-S, using mixed linear regression models with a random intercept using the program R V4.3.3. We provided coefficients, their 95% confidence intervals and *p*-values. The significance level was set at *p* = 0.05.

### 2.4. Missings

At T0, three patients did not reply to the GAD-7, PHQ-9, PHQ-15, FSS and PCS-S; 14 did not reply to PEM; one patient missed two answers in the FSS; and two patients missed one answer in the PCS-S. At T1, two patients missed one answer in the PHQ-9. Missing values were imputed using the random forest imputation with the missForest package.

## 3. Results

*n* = 328 patients, who attended the Post-COVID centre, were invited consecutively for the follow-up. Four patients rejected participation at T1 and 10 patients had to be excluded because their condition was attributed to Post-Vaccination Syndrome. A total of 49 patients did not complete the questionnaires at T1, leading to a response rate of 83.8% and a total number of *n* = 265 included patients.

### 3.1. Sociodemographic and Health-Related Data

The mean age was 45.5 (SD = 12.1) years and the majority of participants were female (*n* = 187, 70.6%) (Table 1). Most participants had graduated from high school (*n* = 128, 48.3%), while 97 (36.6%) had a higher educational certificate. Table 1 only presents baseline data (T0) because they remained very similar at T1 with the exception of employment status. At T0, 88 patients (33.2%) worked full-time and 74 (27.9%) part-time. Upon follow-up (T1), 67 (25.3%) were found to be working full-time and 60 (22.6%) engaged in part-time work. Both the unemployment rate and sick leave/incapacitated for work rate increased over the surveying period from 5.3% to 7.9% and from 15% to 21.9%, respectively.

### 3.2. Post-COVID-Related and Cardiac Variables

The mean time since SARS-CoV-2 infection at follow-up was 22.5 (SD = 8.2) months, with the follow-up surveying period being, on average, 5.0 (SD = 2.4) months. The majority of the sample had suffered a symptomatic COVID-19 infection that did not require inpatient therapy (*n* = 224, 84.5%). Over one-half of the sample had received the vaccination at least three times (*n* = 154, 58.1%) (Table 2).

### 3.3. Mental Health

#### 3.3.1. Severity of Symptoms and Development at Follow-Up (T1)

**Figure 1:**
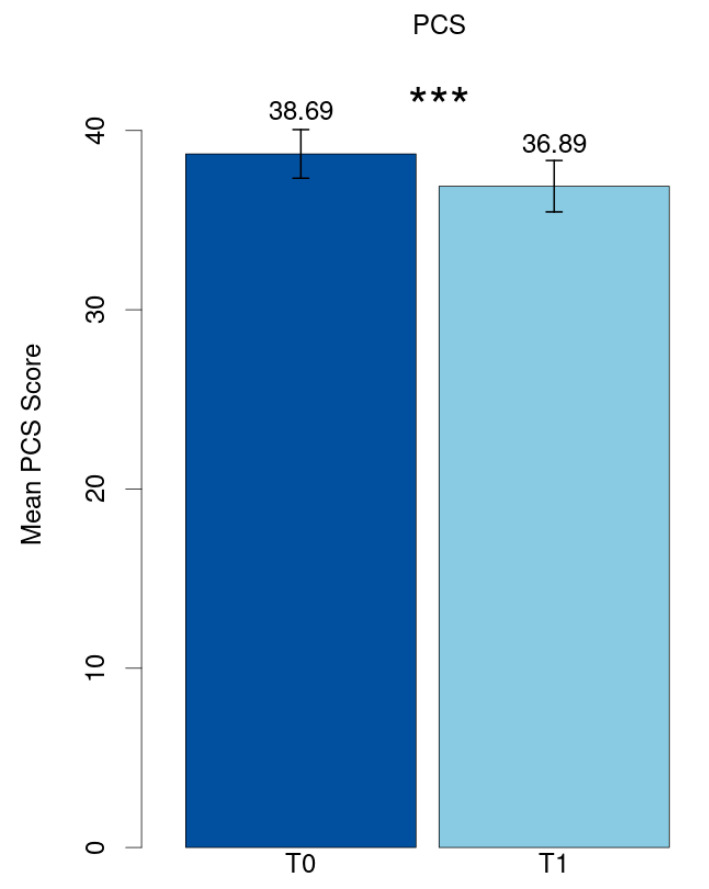
Mean PCS score. *** *p* < 0.001.

The mean value of the Post-COVID Syndrome score at baseline (T0) was 38.69 (SD = 11.21) and reduced significantly to 36.89 (SD = 11.90) at follow-up (T1) (*p* < 0.001, Cohen’s d = 0.21) (Figure 1).

**Figure 2:**
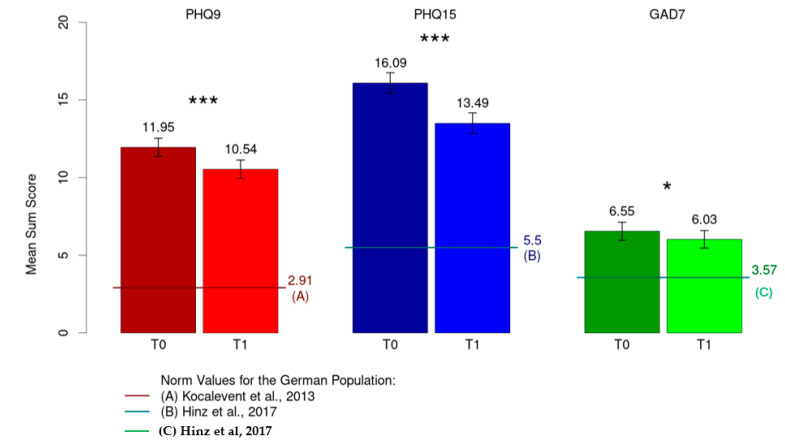
Mean sum score for depression (PHQ-9), somatization (PHQ-15) and anxiety (GAD-7). * *p* < 0.05; *** *p* < 0.001; [38,39,40].

A significant decrease in severity was observed from T0 to T1 for depressive symptoms (M = 11.95, SD = 4.82 to M = 10.54, SD = 4.92; *p* < 0.001, d = 0.34), persisting somatic symptoms (M = 16.09, SD = 5.46 to M = 13.49, SD = 5.60; *p* < 0.001, d = 0.73) and anxiety symptoms (M = 6.55, SD = 4.78 to M = 6.03, SD = 4.69; *p* = 0.038, d = 0.13) (Figure 2).

When compared with the normal population in terms of depression (M = 2.91, SD = 3.52), persistent somatic symptoms (M = 5.5 SD = 3.93) and anxiety (M = 3.57, SD = 3.38) [38,39,40], all three constructs showed a significantly higher mean value in the study sample (*p* < 0.001).

**Figure 3:**
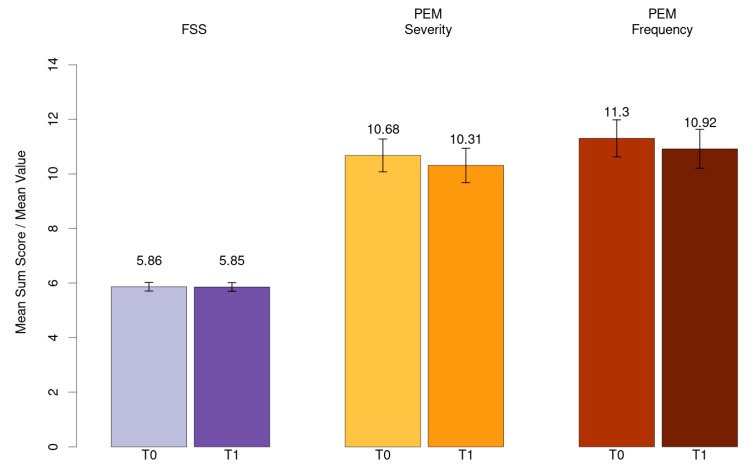
Mean value for fatigue (FSS) and mean sum score for PEM severity and frequency.

Mean value for fatigue (FSS) and mean sum score for PEM severity and frequency has been shown in Figure 3.

#### 3.3.2. Frequency of Symptoms

The frequency of symptoms above the cut-off was calculated at T0 and T1 and is displayed in Table 3. PEM frequency was not calculated due to the absence of a validated cut-off score. The frequency of PASC symptoms, using the PCS-S, is shown in Supplement S2.

### 3.4. Utilization of Psychotherapeutic Treatment

Approximately 30% (*n* = 78) of the patients reported utilization of psychotherapeutic treatment prior to the onset of their PASC condition.

### 3.5. Mixed Model Analysis

Advanced age was associated with higher PHQ-9 values at T1 (*p* < 0.001) (Table 4). A significant reduction in mean scores over time was seen in PHQ-9, PHQ-15, GAD-7 and the PCS-S (p_PHQ.9; PHQ-15, PCS-_s < 0.001; p_GAD-7_ = 0.026). Additionally, a longer time span between the acute infection and baseline was associated with both higher PEM severity and frequency scores at T1 (*p* = 0.038; *p* = 0.017). Other variables such as sex, the course of the acute infection or a pathologic echocardiogram did not reach the level of significance in the analyses.

Patients with mental illness and prior psychotherapy had significantly higher scores in depression, anxiety, somatic and PASC symptoms at T1 compared to those without (p_PHQ-9_ = 0.035, p_PHQ-15_ = 0.006, p_GAD-7; PCS-s_ = 0.038).

## 4. Discussion

To the best of our knowledge, the course of the mental health of PASC patients has, to date, not been comprehensively examined longitudinally. In our study, we present data of participants at our outpatient clinic, using six validated questionnaires (PROMs). Our data provide important insights on the trajectory of the mental health of patients with PASC, as well as on risk and protective factors.

### 4.1. Sociodemographic and Health-Related Variables

Our study sample’s demographics align with prior studies, with mostly female participants averaging 46 years of age [6,8,23]. Education levels were medium to high, and BMI and comorbidity rates resembled previous studies as well [5,23,28,32].

Employment rates at baseline resembled those of Peter’s [28], and, as observed in other studies, our cohort exhibited an increase in sick leave and incapacitation as well as unemployment rates due to PASC [19,28,41].

### 4.2. Post-COVID-Related Variables and Post-COVID Syndrome Score

The mean duration between acute infection and admission to our centre was 17 months extending to 22.5 months at follow-up, longer than in other studies [32]. The surveying period between baseline and follow-up spanned five months, similar to the existing literature [22,23]. However, the standard deviations cover a comparably large time span, which must be considered when comparing mental health changes to other studies.

Most patients received the vaccine three times and suffered a symptomatic course that did not require hospitalization, consistent with the other literature [5,28]. This substantiates the hypothesis that a severe and prolonged course of PASC can be seen regardless of the acute infection course [13,14].

In our sample, severe PASC was seen in 80.8% of the patients at follow-up. A Swedish study [42] revealed a similar prevalence of at least one symptom of PASC after 24 months; another study showed a lower prevalence of 62% [43].

It can be assumed that our patients experienced multiple symptoms, indicating an even higher symptomatic burden than in other studies.

The severity of PASC symptoms reduced significantly over time. However, the mean score was still 36.89, much higher and for a longer period than in other studies. Lemhöfer, for instance, found a mean PCS-S of 23.5 506 days post-infection, significantly lower than in our study cohort [44]. Considering that a score >26.25 indicates severe PASC, it is striking that our patients still experienced profound symptoms 22.5 months after the acute infection. Despite the significant decrease in severity, it remains on a very high level.

It is important to acknowledge that the Post-COVID centre in Erlangen was established specifically for patients already diagnosed with severe PASC. This particular patient population may account for the persistent severity of the symptoms and the heightened symptomatic burden observed in our study compared to other research findings.

### 4.3. Mental Health

#### 4.3.1. Depression, Anxiety, Persisting Somatic Symptoms

Upon follow-up (T1), clinically relevant levels of depression, persisting somatic symptoms and anxiety were prevalent in 55.8%, 72.5% and 18.9% of patients, respectively. These percentages exceed those reported in several other longitudinal studies, particularly considering that many of these studies had follow-up periods of twelve months or less [8,20,28,45]. Guillen-Burgos, surveying patients over 24 months, revealed a lower depression frequency, whereas anxiety stood at a comparable level [25]. The prevalence of these symptoms is much higher in our cohort than reported in previous studies, where the frequency was approximately 20% [26,27,46].

The severity of depressive, anxiety and somatic symptoms exhibited a statistically relevant decrease over the surveying period, which aligns with two recent reviews [16,47]. However, when compared to the normal population, our sample values still significantly surpassed the established cut-off values, even after 22.5 months after infection [38,39,40]. This finding is substantiated in several studies all claiming persistent high symptomatic burdens of depression and anxiety and a general decline in the mental health of PASC patients [15,20,47,48].

Comparing our study’s values to those of other studies with similar sociodemographics reveals comparable to somewhat higher scores [49,50,51].

#### 4.3.2. Fatigue and Post-Exertional Malaise

Fatigue emerges as the most common Post-COVID symptom of our cohort, consistent with numerous studies [4,5,9]. However, severe fatigue’s prevalence in our cohort was much higher than in previous studies [9,26,52,53]. Moreover, fatigue severity did not decrease, supporting the hypothesis that fatigue does not show a self-limiting course and remains on a clinically high level over an extended period. In some cases, it may even worsen over time [10,14,20,48]. Contradictory to our findings, Poole-Wright’s meta-analysis revealed a significant decrease over time [52].

In line with the existing literature, the severity and frequency of PEM showed a similar pattern, lacking a significant decrease over the study’s duration [29,54,55]. It can be assumed that therapy as usual, encompassing the diagnostic process and therapeutic recommendations, did not lead to an alleviation of fatigue and PEM symptoms.

Fatigue and PEM demonstrated a significant correlation in another study, suggesting that most patients suffering from PEM also suffer from ongoing fatigue [55]. It can be assumed that this observation holds true in our study as well, given that both constructs stayed on high levels without improvement over time. Bearing in mind that these symptoms are particularly debilitating for patients in their daily life, it is not striking that the unemployment rates, alongside sick rates, increased over the survey period, underscoring the urgency for special therapeutic options for PASC patients suffering from PEM and fatigue, with the objective of preventing a permanent inability to work and a worsening of the patient’s health over time.

#### 4.3.3. Persistence of Symptoms

The persistence and severity of mental health impairments can potentially be attributed to the mental load caused by severe PASC on individuals striving to return to their pre-infection activity levels. Fatigue and PEM, in particular, pose obstacles to this return, a phenomenon reported in numerous studies and observed within our cohort [19,41,55].

With limited specific therapeutic options available, and no known causal therapy for fatigue and PEM [56], patients may struggle to adapt to ongoing challenges posed by PASC, such as difficulties in resuming activities of daily life. This ongoing incapacitation can lead to feelings of helplessness and patients may feel limited in their ability to improve or seek professional help. This most likely creates a vicious circle that maintains the impairments and the psychological strain of the patients.

Therefore, the slight reduction in symptom severity in some outcomes might result from the diagnostic process and medical attention provided by the Post-COVID centre. Putatively, similar to cancer and other chronic illnesses, confrontation with the new life situation may lead to new flexible forms of coping mechanisms over time, resulting in a reduction in the symptoms of mental health impairment.

The pathophysiological mechanisms of Post-COVID have to be taken into account as well, as systemic and central nervous system inflammation have been described as contributing to depression and anxiety even months after the acute infection [4,47,48]. We did not find a significant association between the course of the acute infection and elevated depressive and anxiety symptoms; however, this might be due to the high number of non-hospitalized patients in our cohort.

### 4.4. Risk and Protective Factors

#### 4.4.1. Sociodemographic Data and Time

In our cohort, advanced age was associated with higher PHQ-9 values at follow-up, consistent with previous studies [4,5,6].

A longer duration between infection and baseline was associated with higher scores in both the severity and frequency of PEM at follow-up. One explanation might be that those patients did not receive guidance on symptom management, such as pacing protocols, early enough. PEM can be exacerbated even with activities of daily life [57], making early recommendations crucial to prevent sustained symptom elevation over time.

Over time, PHQ-9, PHQ-15, GAD-7 and the PCS-S fell significantly. This suggests the hypothesis that some symptoms of PASC may follow a self-limiting course, similar to other post-viral symptoms [31]. A missing association for fatigue and PEM may indicate that these symptoms only diminish slowly or not at all, as is reported in ME/CFS [31].

#### 4.4.2. Psychotherapeutic Utilization before PASC

There was a significant association between higher scores in the PHQ-9, PHQ-15, GAD-7 and PCS-S in patients with a history of mental illness and psychotherapy before the onset of PASC.

This finding aligns with the existing literature indicating that prior psychiatric illnesses are a recognized risk factor for developing mental health symptoms in PASC [4,12,58], as well as more severe symptoms than patients without psychiatric history [59]. Contradictory to our findings, three studies found a protective factor of a prior psychiatric history, leading to lower GAD-7 and PHQ-9 scores in those individuals with previous psychiatric diagnoses than in those without [48,60,61].

### 4.5. Strengths and Limitations

To the best of our knowledge, this study is the first in-depth exploration of the mental health trajectory of Post-COVID patients after the first 22 months of the Post-COVID condition. Notably, this study boasts a substantial cohort size and a prospective design. We utilized six validated questionnaires covering a vast spectrum of possible symptoms and gathered crucial sociodemographic and clinical data as well as the information of prior psychotherapy.

Limitations are, primarily, the absence of a control group. Additionally, potential sex biases may exist, given that 70% of the cohort are females. Furthermore, there might be biases due to the absence of clinical structured interviews; instead, patients self-reported their symptoms and prior illnesses. Lastly, first presentation to the centre was relatively late after the acute infection; therefore, symptom development between the infection and first presentation is unknown.

## 5. Conclusions

In conclusion, this study emphasizes the need for further examination of the long-term mental health outcomes of PASC. The prevalence of severe Post-COVID syndrome was 80%, indicating a substantial symptomatic burden extending for the period of 22 months after infection. Depression, anxiety and somatic symptoms remain prevalent at elevated levels. While their severity exhibits a decrease over time, suggesting a self-limiting course, symptom manifestation is still significantly higher than in the normal population. Notably, symptoms of fatigue and PEM remained unchanged, emphasizing the need for specific therapeutic options for PASC patients and further studies herein. Our findings suggest, that therapy as usual does not lead to a sufficient alleviation of symptoms in fatigue and PEM, whereas other symptoms responded through a slight decrease in severity. Identified risk factors for a more severe disease course encompassed older age for depressive symptoms and a longer duration between infection and presentation at baseline for PEM. Additionally, mentally vulnerable patients, suffering from a prior psychiatric illness, were seen to be associated with more severe symptoms of PASC, depression, anxiety and somatic symptoms.

Further studies including control groups are warranted to assess more risk and potential protective factors and to identify individuals who are more vulnerable to PASC mental health symptoms.

## Figures and Tables

**Table 1:** Sociodemographic and health-related data.

Variables	Sample at Baseline (T0) (*n* = 265)
**Sex, *n* (%)**	
Women	187 (70.6)
Men	75 (28.3)
Missing	3 (1.1)
**Age, years**	
M (SD)	45.5 (12.1)
Range	19–79
**Marital status, *n* (%)**	
Single without partnership	46 (17.4)
Single with partnership	43 (16.2)
Married	157 (59.2)
In separation	2 (0.8)
Widowed	2 (0.8)
Divorced	12 (4.5)
Missing	3 (1.1)
**Children, *n* (%)**	
Yes	161 (60.8)
No	101 (38.1)
Missing	3 (1.1)
**Education level, *n* (%)**	
Without certificate	1 (0.4)
Lower secondary school certificate	44 (16.6)
Secondary school certificate	86 (32.5)
High school certificate	128 (48.3)
Other	3 (1.2)
Missing	3 (1.1)
**Professional qualification, *n* (%)**	
None	13 (4.9)
Master school	54 (20.4)
Apprenticeship certificate	97 (36.6)
University degree	97 (36.6)
Missing	4 (1.5)
**Employment status, *n* (%)**	
Full-time employed	88 (33.2)
Part-time employed	74 (27.9)
Unemployed	14 (5.3)
Retired/pensioned	18 (6.8)
Sick leave/incapacitated for work	40 (15.0)
Other	27 (10.3)
Missing	4 (1.5)
**Smoking, *n* (%)**	
Yes	19 (7.2)
No	238 (89.8)
Missing	8 (3.0)
**BMI, kg/m^2^**	
M (SD)	26.2 (5.4)
Range	16.6–49.6
<18.5 (*n*, %)	9 (3.4)
18.5–24.9 (*n*, %)	116 (43.8)
≥25–29.9 (*n*, %)	80 (30.2)
>30–39.9 (*n*, %)	53 (20.0)
>40 (*n*, %)	4 (1.5)
Missing	3 (1.1)
**Self-reported pre-existing disease, *n* (%) ^1^**	
Yes	112 (42.3)
No	153 (57.7)
Hormonal or metabolic	37 (14)
Psychiatric diseases	25 (9.4)
Nervous system	7 (2.6)
Cardiovascular system	18 (6.8)
Respiratory system	28 (10.6)
Musculoskeletal and connective tissue	24 (9.1)
Digestive system	20 (7.5)
Dermatological	16 (6.0)
Other	32 (12.1)

^1^ Raised at T1.

**Table 2:** Post-COVID-Related and cardiac data.

Variables	Sample at Follow-Up (T1) (*n* = 265)
**Time between the SARS-CoV-2 infection and baseline (T0), months**	
M (SD)	17.1 (8.7)
Range	3–42
**Time between the SARS-CoV-2 infection and follow-up (T1), months**	
M (SD)	22.5 (8.2)
Range	10–47
**Time between baseline (T0) and follow-up (T1), months**	
M (SD)	5.0 (2.4)
Range	3–11
**Course of the acute SARS-CoV-2 infection, *n* (%)**	
Asymptomatic	14 (5.3)
Symptomatic, therapy at home or outpatient	224 (84.5)
Symptomatic, inpatient therapy without intensive care admission	21 (7.9)
Symptomatic, inpatient therapy with intensive care admission	6 (2.3)
**PCR, *n* (%)**	
Carried out	236 (89.1)
Not carried out	29 (10.9)
**Vaccine, *n* (%)**	
None	14 (5.3)
1×	11 (4.2)
2×	58 (21.9)
3×	154 (58.1)
4×	24 (9.1)
5×	4 (1.5)
**Cardiac pathology, *n* (%)**	
Yes (Scores 1–3)	28 (10.6)
No (Score 0)	197 (74.3)
Missing	40 (15.1)
Ejection fraction	3 (1.1)
Pulmonic artery pressure elevation	5 (1.9)
Left ventricle dilatation	9 (3.4)
Pericardial effusion present	13 (4.9)
Atrial fibrillation	0 (0.0)

**Table 3:** Frequency of symptoms.

Variables	Sample at Baseline (T0) (*n* = 265) ^1^	Sample at Follow-Up (T1) (*n* = 265)
**Post-COVID Symptom Score (PCS-S)**, cut-off >26.25		
*n* (%)	224 (84.5)	214 (80.8)
**Fatigue Severity Scale (FSS)**, cut-off ≥4		
*n* (%)	237 (89.4)	237 (89.4)
**Patient Health Questionnaire-9 (PHQ-9)**, cut-off ≥10		
*n* (%)	181 (68.3)	148 (55.8)
**Generalized Anxiety Disorder-7 (GAD-7)**, cut-off ≥10		
*n* (%)	64 (24.2)	50 (18.9)
**Patient Health Questionnaire-15 (PHQ-15)**, cut-off >10		
*n* (%)	230 (86.8)	192 (72.5)

**^1^** Sample size may vary in the different questionnaires due to missings.

**Table 4:** Mixed model analysis.

	Variable (T1)			
Outcome Measure (Coefficient, [95% Confidence Interval], *p*-Value)	PHQ-9 ^1^	PHQ-15 ^2^	GAD-7 ^3^	FSS ^4^
Intercept	7.830, [4.104; 11.556], <0.001 *	17.206, [12.929; 21.483], **<0.001**	4.748, [1.274; 8.222], **0.008**	5.102, [4.12; 6.084], **<0.001**
Sex (Male)	0.120, [−1.20; 1.441], 0.858	−1.321, [−2.847; 0.205], 0.091	0.318, [−0.911; 1.547], 0.612	0.001, [−0.346; 0.349], 0.995
Age	0.064, [0.011; 0.117], **0.018**	−0.003, [−0.063; 0.058], 0.927	0.027, [−0.022; 0.076], 0.284	0.009, [−0.005; 0.023], 0.216
BMI ≥25	0.371, [−0.777; 1.519], 0.527	−0.532, [−1.758; 0.694], 0.396	0.096, [−0.001; 1.184], 0.862	−0.173, [−0.481; 0.134], 0.27
Smoker	−0.889, [−2.567; 0.789], 0.3	−1.425, [−3.2; 0.35], 0.116	−0.767, [−2.316; 0.827], 0.346	−0.093, [−0.543; 0.357], 0.685
Pathologic echocardiography	−0.409, [−2.22; 1.404], 0.659	0.219, [−1.88; 2.317], 0.839	0.545, [−1.14; 2.23], 0.527	−0.274, [−0.751; 0.203], 0.261
Working	−0.806, [−1.924; 0.312], 0.659	−0.453, [−1.537; 0.631], 0.413	−0.430, [−1.518; 0.658], 0.439	−0.234, [−0.539; 0.071], 0.133
Not working	−0.630, [−1.848; 0.588], 0.311	0.102, [−1.077; 1.281], 0.866	−0.382, [−1.569; 0.804], 0.528	−0.077, [−0.412; 0.258], 0.651
Prior psychiatric illness without prior psychotherapy	−3.641, [−8.752; 1.471], 0.164	−2.604, [−8.523; 3.314], 0.389	−0.386, [−5.139; 4.366], 0.874	0.047, [−1.299; 1.393], 0.945
Prior psychiatric illness with prior psychotherapy	2.370, [0.186; 4.554], **0.035**	3.566, [1.039; 6.039], **0.006**	2.159, [0.128; 4.191], **0.038**	0.424, [−0.151; 1], 0.15
Partnership (Yes)	0.710, [−0.513; 1.933], 0.256	0.995, [−0.301; 2.291], 0.133	0.284, [−0.877; 1.444], 0.632	−0.155, [−0.483; 0.174], 0.357
Time between the acute infection and T0	<0.001, [−0.75; 0.076], 1	0.024, [−0.063; 0.112], 0.586	−0.011, [−0.081; 0.06], 0.765	0.013, [−0.007; 0.033], 0.207
High school certificate	−0.603, [−1.841; 0.635], 0.341	−1.178, [−2.604; 0.249], 0.107	−0.543, [−1.696; 0.609], 0.357	0.035, [−0.291; 0.36], 0.834
Acute infection with inpatient treatment	0.080, [−1.899; 2.059], 0.937	0.898, [−1.396; 3.192], 0.444	1.278, [−0.561; 3.118], 0.175	−0.213, [−0.734; 0.308], 0.424
Number of vaccinations	0.364, [−0.287; 1.016], 0.275	−0.287, [−1.042; 0.468], 0.457	0.165, [−0.441; 0.771], 0.595	0.141, [−0.031; 0.312], 0–109
Time to T1	−1.613, [−2.158; −1.069], **<0.001**	−2.716, [−3.209; −2.223], **<0.001**	−0.621, [−1.164; −0.078], **0.026**	−0.045, [−0.198; 0.108], 0.565
**Outcome Measure (Coefficient, 95% Confidence Interval, *p*-Value)**	**PCS-S ^5^**	**PEM Frequency ^6^**	**PEM Severity ^6^**	
Intercept	34.203, [25.427; 42.978], **<0.001**	8.719, [4.25; 13.189], **<0.001**	9.262, [5.369; 13.154], **<0.001**	
Sex (Male)	−2.762, [−5.883; 0.359], 0.084	0.408, [−1.187; 2.004], 0.616	0.227, [−1.163; 1.617], 0.749	
Age	0.095, [−0.03; 0.219], 0.138	−0.004, [−0.068; 0.061], 0.91	−0.013, [−0.069; 0.044], 0.661	
BMI ≥25	−0.915, [3.565; 1.734], 0.499	−0.451, [−1.817; 0.916], 0.518	0.045, [−1.137; 1.228], 0.94	
Smoker	−0.530, [−4.388; 3.328], 0.788	−0.439, [−2.54; 1.662], 0.682	−0.160, [−1.98; 1.659], 0.863	
Pathologic echocardiography	−1.554, [−5.843; 2.734], 0.478	−1.327, [−3.522; 0.867], 0.237	−0.707, [−2.619; 1.206], 0.47	
Working	−1.847, [−4.318; 0.623], 0.144	−0.516, [−1.763; 0.731], 0.418	−0.549, [−1.617; 0.518], 0.314	
Not working	1.103, [−1.612; 3.818], 0.427	0.235, [−1.141; 1.611], 0.738	0.462, [−0.717; 1.641], 0.443	
Prior psychiatric illness without prior psychotherapy	−3.446, [−15.541; 8.648], 0.577	2.068, [−4.099; 8.236], 0.512	1.747, [−3.629; 7.123], 0.525	
Prior psychiatric illness with prior psychotherapy	5.509, [0.342; 10.676], **0.038**	1.462, [−1.167; 4.092], 0.277	1.421, [−0.871; 3.713], 0.226	
Partnership (Yes)	−0.227, [−3.046; 2.592], 0.875	−0.066, [−1.497; 1.365], 0.928	−0.240, [−1.477; 0.997], 0.704	
Time between the acute infection and T0	0.131, [−0.047; 0.309], 0.151	0.112, [0.021; 0.203], **0.017**	0.085, [0.005; 0.164], **0.038**	
High school certificate	−0.522, [−3.441; 2.397], 0.726	−0.499, [−1.993; 0.995], 0.513	−0.740, [−2.042; 0.561], 0.266	
Acute infection with inpatient treatment	−2.500, [−7.184; 2.183], 0.297	−1.559, [−3.943; 0.826], 0.202	−1.267, [−3.345; 0.812], 0.234	
Number of vaccinations	0.014, [−1.527; 1.554], 0.986	0.378, [−0.407; 1.162], 0.347	0.298, [−0.386; 0.982], 0.394	
Time to T1	−2.233, [−3.413; −1.054], **<0.001**	−0.422, [−1.017; 0.174], 0.167	−0.425, [−0.959; 0.055], 0.082	

* Significant *p*-values are marked in bold. ^1^ Patient Health Questionnaire-9. ^2^ Patient Health Questionnaire-15. ^3^ Generalized Anxiety Disorder-7. ^4^ Fatigue Severity Scale. ^5^ Post-COVID Syndrome Score. ^6^ DePaul Post-Exertional Malaise Screening.

## Data Availability

The data used for the current study are not publicly available due to ethical and legal data protection restrictions.

## Supplementary Materials

The following supporting information can be downloaded at: https://www.mdpi.com/article/10.3390/ijerph21081076/s1, Supplement S1: Algorithm for echocardiograms; Supplement S2: PASC symptoms (PCS-S) at T0 and T1.
