# Supplementary material for: Three-Month Follow-Up of the Post-COVID Syndrome after Admission to a Specialised Post-COVID Centre—A Prospective Study Focusing on Mental Health with Patient Reported Outcome Measures (PROMs)
Source: Int J Environ Res Public Health. 2024 Aug 16;21(8):1076. doi: 10.3390/ijerph21081076 (PMC11354797; doi:10.3390/ijerph21081076)
Supplement: Supplementary file 1 [file ijerph-21-01076-s001.zip › ijerph-3144262-supplementary.pdf]

Supplement S1: Algorithm for echocardiograms

**Variables**	**Pathology**	**Score**
Ejection fraction	Normal	0
	Mild restriction (>50%)	1
	Moderate restriction (40-49%)	2
	Severe restriction (<40%)	3
Left atrium size	No dilatation	0
	Dilatation	1
Pulmonic artery pressure	Normal (15-25mmHg)	0
	Slight pressure elevation/elevation probable	1
	Definitive pressure elevation >28mmHg	2
Pericardial effusion	None	0
	Present	1
Atrial fibrillation	None	0
	Present	1

**Total points**	**Sum Score**	**Interpretation**
0	0	No cardiac pathology
1	1	Mild cardiac pathology
2	2	Moderate cardiac pathology
3 – 8	3	Severe cardiac pathology

Supplement S2: PASC symptoms (PCS-S)[1] at T0 and T1

**Symptoms, *n (%)***	**Present at T0 (n=265)**	**Present at T1 (n=265)**
Ageusia or anosmia	42 (15.8)	40 (15.1)
Fatigue	250 (94.3)	241 (90.9)
Lack of physical resilience	251 (94.7)	235 (88.7)
Joint or muscle pain	202 (76.2)	199 (75.1)
Throat/nose/ear discomfort	123 (46.4)	125 (47.2)
Lung /breathing difficulties	122 (46.0)	104 (39.2)
Cardiac symptoms	157 (59.2)	153 (57.7)
Intestinal symptoms	128 (48.3)	123 (46.4)
Neurological complaints	249 (94.0)	242 (91.3)
Dermal problems	112 (42.3)	99 (37.4)
Signs of infection	86 (32.5)	91 (34.3)

Sleeping disorders	212 (80.0)	212 (80.0)

[1] Post-COVID-Syndrome Score
